# Japanese-Style Diet and Cardiovascular Disease Mortality: A Systematic Review and Meta-Analysis of Prospective Cohort Studies

**DOI:** 10.3390/nu14102008

**Published:** 2022-05-10

**Authors:** Masayuki Shirota, Norikazu Watanabe, Masataka Suzuki, Masuko Kobori

**Affiliations:** 1 Institute of Food Research, National Agriculture and Food Research Organization, 2-1-2 Kannondai, Tsukuba-shi 305-8642, Ibaraki, Japan; shirotam620@affrc.go.jp; 2 Yakujihou Marketing Jimusho Inc., Level 3, Sanno Park Tower, 2-11-1 Nagata-cho, Chiyoda-ku, Tokyo 100-6162, Japan; watanabe@yakujihou-marketing.co.jp (N.W.); suzuki@yakujihou-marketing.co.jp (M.S.)

**Keywords:** Japanese dietary pattern, Japanese diet, mortality, cardiovascular disease, systematic review, meta-analysis

## Abstract

This systematic review and meta-analysis elucidate the effects of the Japanese-style diet and characteristic Japanese foods on the mortality risk of cardiovascular disease (CVD), cerebrovascular disease (stroke), and heart disease (HD). This review article followed the PRISMA guidelines. A systematic search in PubMed, The Cochrane Library, JDreamIII, and ICHUSHI Web identified prospective cohort studies on Japanese people published till July 2020. The meta-analysis used a random-effects model, and heterogeneity and publication bias were evaluated with I^2^ statistic and Egger’s test, respectively. Based on inclusion criteria, we extracted 58 articles, including 9 on the Japanese-style diet (*n* = 469,190) and 49 (*n* = 2,668,238) on characteristic Japanese foods. With higher adherence to the Japanese-style diet, the pooled risk ratios (RRs) for CVD, stroke, heart disease/ischemic heart disease combined (HD/IHD) mortality were 0.83 (95% CI, 0.77–0.89, I^2^ = 58%, Egger’s test: *p* = 0.625, *n* = 9 studies), 0.80 (95% CI, 0.69–0.93, I^2^ = 66%, Egger’s test: *p* = 0.602, *n* = 6 studies), and 0.81 (95% CI, 0.75–0.88, I^2^ = 0%, Egger’s test: *p* = 0.544, *n* = 6 studies), respectively. Increased consumption of vegetables, fruits, fish, green tea, and milk and dairy products decreased the RR for CVD, stroke, or HD mortality. Increased salt consumption elevated the RR for CVD and stroke mortality. Increased consumption of dietary fiber and plant-derived protein decreased the RR for CVD, stroke, and HD/IHD mortality. The Japanese-style diet and characteristic Japanese foods may reduce CVD mortality. Most studies conducted diet surveys between 1980 and the 1990s. This meta-analysis used articles that evaluated the same cohort study by a different method. A new large-scale cohort study matching the current Japanese dietary habits is needed to confirm these findings.

## 1. Introduction

There have been several prospective cohort studies of cardiovascular disease (CVD) risk. In the Seven Countries Study [1,2,3,4] and Framingham Study [5], the authors reported that (1) blood pressure and serum total cholesterol concentration are positively related to the mortality of ischemic heart disease (IHD), and cerebrovascular disease (stroke), (2) HDL-cholesterol concentration is inversely associated with IHD mortality, and (3) LDL-cholesterol concentration is positively associated with IHD mortality.

In Japan, the stroke mortality rate was the highest among all-cause mortality from 1951 to 1980 [6]. Therefore, prospective cohort studies were initiated on stroke mortality, especially cerebral hemorrhage, in a particular district [7,8,9,10,11]. These studies reported that blood pressure was the highest risk factor for cerebral hemorrhage, and this mortality rate declined conspicuously from the 1970s onward. The causes of this decline were sufficient blood pressure control with hypertensive drugs and beneficial changes in nutrition and lifestyle.

Beginning in the 1980s, large cohort studies on a nationwide scale were initiated. They investigated the relationship between cause-specific risk and diet pattern/food intake. The results suggested that Japanese-style dietary patterns and characteristic Japanese food intake would contribute to disease prevention and lifespan extension. Based on the results of the dietary survey, we evaluate the Japanese-style diet from the dietary pattern score calculated by factor analysis and the index score calculated from the intake status of characteristic foods. The characteristic Japanese foods in this review are vegetables, fruits, fish, soy products, green tea, seaweed, pickles, rice, and meat used to calculate the Japanese food index score. Suzuki et al. [12] reported that the Japanese-style diet’s characteristic foods consisted of soybeans/soybean products, seafood, vegetables, rice, and miso soup. Moreover, Tsugane [13] mentioned that the Japanese people consumed less meat, milk/dairy products, sweeteners, and fruits but more fish/seafood, soybeans, and green tea. These characteristics of the Japanese-style diet are in partly similar to those of the Mediterranean diet [14,15], which is widely known to reduce CVD risk [15,16,17,18,19,20,21].

Over the past six decades, carbohydrate intake has decreased, and fat intake has increased dramatically, considering the changes in nutrition [22]. In addition, the consumption of rice, vegetable, fish, and soybean products has decreased, but that of meats and dairy products has increased [23]. Concurrently with the nutritional changes, the average longevity of Japanese people has extended noticeably [24].

Under these circumstances, the Japanese diet has attracted attention outside Japan due to growing health consciousness, and the Japanese traditional food culture “Washoku” was registered as a UNESCO World Heritage in 2013. Despite declining stroke mortality, CVD mortality remains high, accounting for approximately 25% of all deaths [6]. As the characteristics of a Japanese-style diet are similar to those of a Mediterranean diet, we could anticipate a reduction in CVD risk. Therefore, we conducted a qualitative systematic review and meta-analysis of prospective cohort studies conducted in Japan on the association between CVD mortality and Japanese-style dietary patterns or Japanese characteristic food intake.

## 2. Methods

The review protocol was developed for this systematic review and meta-analysis but not registered. This article followed the PRISMA guidelines (Table S1).

### 2.1. Search Strategy

An electronic bibliographic search was conducted in PubMed (the National Library of Medicine, Bethesda, MD, USA), The Cochrane Library (Cochrane, London, UK), JDreamIII (G-Search Limited, Tokyo, Japan), and ICHUSHI Web (Japan Medical Abstract Society, Tokyo, Japan) to identify related studies published in English and Japanese till July 2020. The search was performed using the following combinations of keywords and phrases: “Japan” or “Japanese” or “meal” or “food” or “Japanese food” or “cohort study” or “prospective study” or “mortality” (Table S2).

### 2.2. Eligibility Criteria

The following relevant articles were included in this systematic review: (1) original references, except for reviews, meta-analyses, editorials, conference abstracts, and nonresearch letters, (2) articles that involved general adult populations in Japan, (3) prospective cohort studies, (4) studies that examined the association between Japanese-style diets/characteristic Japanese foods and CVD mortality, and (5) studies that included odds ratio, risk ratio (RR), or hazard ratio for the mortality of CVD, stroke, heart disease (HD), and IHD.

### 2.3. Study Selection

Two reviewers independently evaluated the titles and abstracts of all retrieved articles in the initial search. Articles not meeting the eligibility criteria were excluded using a hierarchical approach based on population, study design, diet styles and patterns, and outcome. We also surveyed the citations and bibliographies of the retrieved articles for other potentially relevant articles by hand-searching. Any disagreements were discussed and resolved by consensus or by a third independent reviewer.

### 2.4. Data Extraction

Two reviewers independently performed data extraction from the included articles. Discrepancies were discussed and resolved by consensus or by a third independent reviewer. The following information was extracted: first author, publication year, study design, sample size, sex, dietary assessment method, method of identifying Japanese dietary pattern (i.e., factor analysis or index score method), Japanese dietary pattern, characteristic Japanese foods (vegetables, fruits, fish, soybean and soybean products, green tea, seaweed, pickles, rice, etc.), RR for the mortality of CVD, and adjusted confounders. We included articles that evaluated the dietary patterns using different methods in the same cohort study.

We considered the characteristic Japanese foods by referring to factor-loading values by factor analysis and foods used to calculate index score.

### 2.5. Quality Assessment

Based on *Minds Manual for Guideline Development* [25,26], two reviewers evaluated the quality of included articles through primary and secondary screening in the following eight items: (1) selection bias, (2) performance bias, (3) detection bias, (4) attrition bias, (5) other bias, (6) indirectness, (7) imprecision, and (8) upgrade factor. Each item, excluding the upgrade factor, was rated as three levels, viz., high (−2), moderate/unclear (−1), and low (0). The summaries reflected the body of evidence at the following three levels: high (−2), moderate (−1), and low (0).

As the upgrade factor, we evaluated the magnitude of effect, plausible confounders, and dose–response gradient. We rated as +1, respectively, the magnitude of effect when both RR and the upper limit of 95% CI were <1, the plausible confounders when unadjusted confounders might be working to diminish the estimated effect, and the dose–response gradient when the P-trend for RR was significant (*p* < 0.05). We also evaluated the summary of the upgrade factor as +1 when two or more of the above three items correspond to +1.

Based on the GRADE (Grading of Recommendations, Assessment, Development, and Evaluation) approach [25,26,27], we additionally evaluated the evidence of body across the studies for each outcome in the following seven items: (1) risk of bias, (2) indirectness, (3) imprecision, (4) inconsistency, (5) other bias (publication bias, etc.), (6) upgrade factor, and (7) strength of evidence. Each item, excluding the upgrade factor and strength of evidence, was rated as three levels, viz., high (−2), moderate/unclear (−1), and low (0). Imprecision was evaluated as −1 (moderate/unclear) or −2 (high) when the total relative weights of studies that rated imprecision as −1 or −2 exceeded 50% in a meta-analysis. Inconsistency was rated as −1 (moderate/unclear) or −2 (high), depending on the distribution of point positions and 95% CI overlap, if I^2^ was >50%. Publication bias was evaluated as −1 (moderate/unclear) or −2 (high) depending on the dispersion of distribution on the Funnel plot when Egger’s test [28] was significant (*p* < 0.1). Furthermore, in a meta-analysis, the upgrade factor was rated as +1 if the sum of the relative weights of studies with an upgrade factor summary of +1 exceeded 50%. Strength of evidence was evaluated as four levels, viz., high, moderate, low, and very low, and was downgraded or upgraded from low, comprehensively judging the score of each evaluation item. Discrepancies were discussed and resolved by consensus or by a third independent reviewer.

### 2.6. Statistical Analysis

The dietary pattern results evaluated CVD mortality risk using the different quantiles of the dietary pattern score or index score. The characteristic foods results evaluated CVD mortality risk using the different quantiles of intake or ingested frequency. We conducted a meta-analysis to evaluate the risk of CVD mortality in the highest compared with the lowest quantile. A meta-analysis used the hazard ratio adjusted for the most variables for confounding factors. The odds ratio or risk ratio also was regarded as a measure of relative risk, and the ratio adjusted for the most confounding factor variables was used. Multivariable-adjusted RRs with 95% confidence intervals from individual studies were combined to calculate an overall RR. We used random-effects models for the analysis and determined heterogeneity using the I^2^ statistics. We tested publication biases using Egger’s test (significant at *p* < 0.1). The meta-analysis was analyzed using Comprehensive Meta-Analysis (Ver.3.3.070, 2014) from Biostat Inc. Moreover, if not otherwise specified, *p* values of <0.05 were considered as significant.

## 3. Results

### 3.1. Study Selection

**Figure 1:**
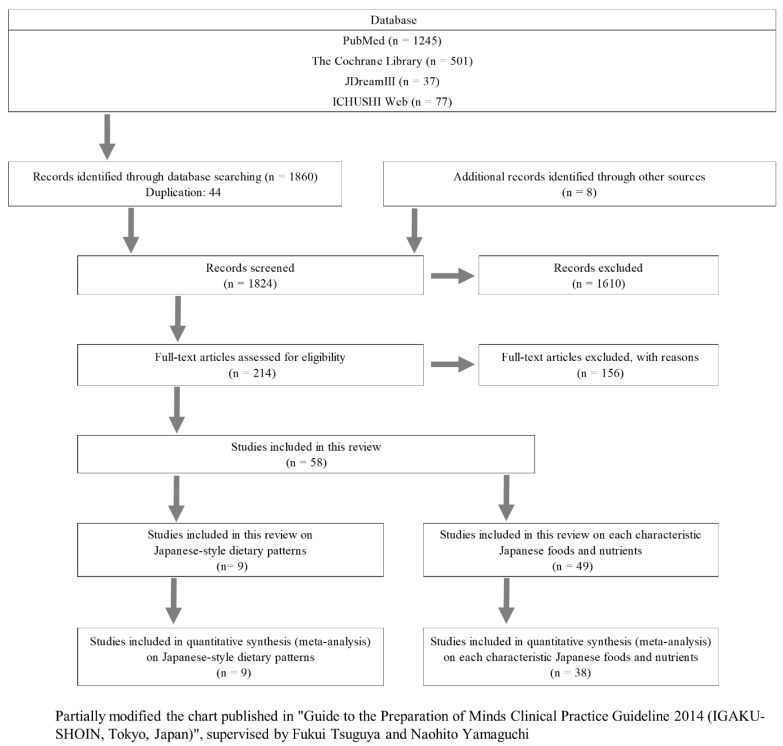
Flowchart of article search and study selection: PubMed (https://pubmed.ncbi.nlm.nih.gov/ (accessed on 27 July 2020)), The Cochrane Library (https://www.cochranelibrary.com/ (accessed on 27 July 2020)), JDreamIII (https://jdream3.com/ (accessed on 27 July 2020)) and ICHUSHI Web (https://search.jamas.or.jp/ (accessed on 27 July 2020)).

Figure 1 shows the flowchart illustrating the process of study selection. A literature search identified 1860 articles and excluded 1802 articles. Finally, 58 articles met the eligibility criteria (Figure 1, Table 1 and Table S3). We used 9 and 38 articles on Japanese dietary patterns and characteristic foods, respectively, in this meta-analysis. The subjects in all studies were adult Japanese people.

We used the Japanese diet index score (index score methods) and Japanese-style dietary pattern score (factor analysis methods) as adherent scores to the Japanese-style diet. All studies divided subjects into three to five quantiles. We used the highest score quantile as the intervention group and the lowest score quantile as the control group.

Concerning the characteristic foods, i.e., carbohydrates (rice, cereal), soybeans, soybean products (miso soup, tofu), seaweeds, pickles, vegetables (including only green-yellow vegetables), fish (fresh fish, roasted fish, boiled fish), green tea, fruits, beans, saturated fatty acids, salt (sodium), dietary GI, dietary GL, animal proteins (meat, eggs, milk products), and plant-derived proteins (soy, vegetables), the intake and ingested frequency were divided into two from five quantiles, respectively. We used the highest consumption group as the intervention group.

### 3.2. Quality Assessment

The risk of bias, indirectness, imprecision, and upgrade factor were evaluated for each outcome of the article (Table S4). Risk of bias and indirectness were 0 (low) for each endpoint as they did not affect the study results. As the number of subjects between the intervention and control groups differed in several articles included in our meta-analysis, we evaluated the imprecision of those reports as −1 (moderate/unclear).

Regarding the body of evidence, we evaluated the risk of bias, indirectness, imprecision, inconsistency, other (publication bias, etc.), upgrade factor, and strength of evidence (Table S5).

### 3.3. Results of Individual Studies, Results of Syntheses, and Additional Analyses

We recruited 9 and 49 studies evaluating the association between Japanese-style dietary patterns or consumption of characteristic Japanese foods and the risk of CVD mortality, respectively.

#### 3.3.1. Japanese-Style Dietary Patterns and Risk of CVD Mortality

The nine articles concerning the Japanese-style dietary pattern and the risk of CVD mortality evaluated the following five cohort studies: Ohsaki Cohort 1994 study (Ohsaki Cohort 1994), NIPPON DATA80, Takayama Study, Japan Collaborative Cohort Study (JACC), and Japan Public Health Center-based Prospective Study (JPHC) (Table 1). Of these nine articles, three evaluated Japanese dietary pattern scores by factor analysis [29,30,31], and six articles evaluated Japanese dietary index scores by the index methods. Of the six articles [32,33,34,35,36,37], two [33,34] evaluated the scores using JFGS (Japanese Food Guide Score), two [36,37] evaluated the scores using JDI (Japanese Diet Index) or its modification (JDI-8), one [35] evaluated the scores using JFS (Japanese Food Score), and one [32] evaluated the scores using low-salt Japanese diet score (Reduced-Salt Japanese Diet Score).

**Figure 2:**
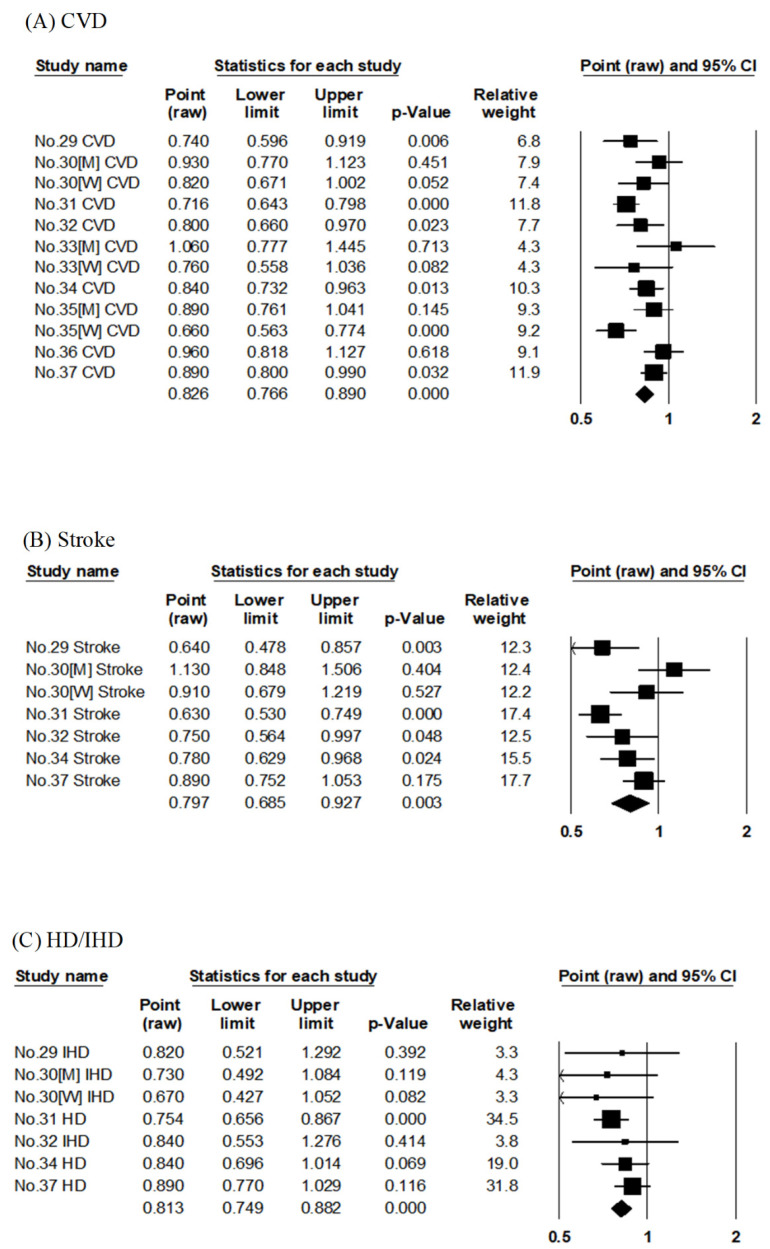
Meta-analysis of the Japanese-style dietary pattern and mortality risk. (**A**–**C**) show the association between the Japanese-style dietary pattern and CVD, stroke, and HD/IHD mortality risk, respectively. The area of each square is proportional to the study weight. Horizontal lines represent 95% confidence intervals. Diamonds represent pooled estimates from inverse-variance-weighted random-effects model. The number given in the study name indicates that of the cited reference [29,30,31,32,34,37]. CVD; cardiovascular disease, HD/IHD; heart disease/ischemic heart disease.

In JACC, the association between the risk of CVD mortality was evaluated using dietary pattern score by factor analysis [30] and JFS [35], albeit with different follow-up periods. Similarly, in Osaki Cohort 1994, factor analysis [29] and JDI [36] were used; in JPHC, factor analysis [31], JFGS [34], and JDI-8 [37] were used to evaluate the association with the risk of CVD mortality. The pooled RR for CVD mortality compared with the highest to lowest adherence to Japanese-style diet was 0.83 (95% CI: 0.77–0.89, *p* < 0.001, I^2^ = 58%, Egger’s test: *p* = 0.625), as shown in Table 2 and Figure 2A. The Japanese-style diet was significantly associated with reduced RR for CVD mortality.

**Table 2:** List of meta-analysis results on CVD, stroke, and HD/IHD mortality risk.

Evaluation Item	Outcome	No. of Studies	No. of Participants	Pooled RR (95%CI)	*p*-Value	I^2^ (%)	Egger’s Test (*p*-Value)	Strength of Evidence *
Japanese-style dietary pattern	CVD	9	468,740	0.83 (0.77–0.89)	*p* < 0.001	58	*p* = 0.625	Moderate ⊕⊕⊕⊖
	Stroke	6	367,953	0.80 (0.69–0.93)	*p* = 0.003	66	*p* = 0.602	Moderate ⊕⊕⊕⊖
	HD/IHD	6	367,953	0.81 (0.75–0.88)	*p* < 0.001	0	*p* = 0.544	Low ⊕⊕⊖⊖
Vegetable	CVD	4	47,306	0.85(0.76–0.96)	*p* = 0.009	21	*p* = 0.138	Low ⊕⊕⊖⊖
	Stroke	5	418,428	0.89(0.80–1.001)	*p* = 0.053	52	*p* = 0.185	Low ⊕⊕⊖⊖
	HD/IHD	3	156,821	0.79 (0.69–0.90)	*p* < 0.001	0	*p* = 0.349	Low ⊕⊕⊖⊖
Fruit	CVD	5	149,801	0.85 (0.79–0.91)	*p* < 0.001	19	*p* = 0.296	Low ⊕⊕⊖⊖
	Stroke	3	107,034	0.70 (0.63–0.77)	*p* < 0.001	0	*p* = 0.605	Moderate ⊕⊕⊕⊖
Fish	CVD	3	110,097	0.86 (0.78–0.94)	*p* < 0.001	28	*p* = 0.082	Very low ⊕⊖⊖⊖
	Stroke	4	327,151	0.87 (0.81–0.93)	*p* < 0.001	0	*p* = 0.099	Very low ⊕⊖⊖⊖
	IHD	3	108,429	0.88 (0.66–1.19)	*p* = 0.409	0	*p* = 0.380	Low ⊕⊕⊖⊖
Soy Pruducts	CVD	4	192,545	0.94 (0.87–1.02)	*p* = 0.137	0	*p* = 0.640	Low ⊕⊕⊖⊖
Green tea	CVD	3	135,436	0.59 (0.38–0.92)	*p* = 0.02	83	*p* = 0.182	Very low ⊕⊖⊖⊖
	Stroke	3	214,099	0.76 (0.63–0.92)	*p* = 0.005	28	*p* = 0.691	Low ⊕⊕⊖⊖
	HD/IHD	3	214,099	0.75 (0.65–0.88)	*p* < 0.001	1	*p* = 0.515	Low ⊕⊕⊖⊖
Milk and dairy products	CVD	3	147,233	0.93 (0.87–0.998)	*p* < 0.045	37	*p* = 0.963	Low ⊕⊕⊖⊖
	Stroke	3	306,673	0.81 (0.75–0.88)	*p* < 0.001	17	*p* = 0.583	Very low ⊕⊖⊖⊖
Rice	Stroke	3	202,837	1.02 (0.89–1.17)	*p* = 0.807	0	*p* = 0.808	Low ⊕⊕⊖⊖
Meat	CVD	3	311,983	0.91 (0.76–1.09)	*p* = 0.319	63	*p* = 0.069	Very low ⊕⊖⊖⊖
Salt	CVD	4	161,337	1.18 (1.03–1.34)	*p* = 0.013	63	*p* = 0.243	Low ⊕⊕⊖⊖
	Stroke	3	152,222	1.30 (1.16–1.46)	*p* < 0.001	0	*p* = 0.358	Very low ⊕⊖⊖⊖
	IHD	3	152,222	0.99 (0.76–1.29)	*p* = 0.929	59	*p* = 0.065	Very low ⊕⊖⊖⊖
Plant-derived protein	CVD	3	107,519	0.81(0.71–0.92)	*p* = 0.001	0	*p* = 0.772	Low ⊕⊕⊖⊖
	Stroke	4	136,598	0.75 (0.64–0.89)	*p* = 0.001	0	*p* = 0.967	Low ⊕⊕⊖⊖
	HD/IHD	3	107,519	0.75 (0.59–0.95)	*p* = 0.015	0	*p* = 0.696	Moderate ⊕⊕⊕⊖
Dietary fiber	CVD	3	160,579	0.77 (0.71–0.84)	*p* < 0.001	0	*p* = 0.794	Moderate ⊕⊕⊕⊖
	Stroke	3	160,579	0.84 (0.73–0.98)	*p* = 0.023	19	*p* = 0.941	Low ⊕⊕⊖⊖
	IHD	3	160,579	0.76 (0.69–0.85)	*p* < 0.001	0	*p* = 0.665	Moderate ⊕⊕⊕⊖
Saturated fatty acid	Stroke	3	145,481	0.95 (0.84–1.07)	*p* = 0.365	34	*p* = 0.350	Low ⊕⊕⊖⊖

CVD; cardiovascular disease, HD; heart disease, IHD; ischemic heart disease, HD/IHD; HD and IHD combined associated with reduced RR for CVD mortality. * Strength of evidence was rated as four levels, viz., high ⊕⊕⊕⊕; moderate ⊕⊕⊕⊖; low ⊕⊕⊖⊖; and very low ⊕⊖⊖⊖.

#### 3.3.2. Japanese-Style Dietary Patterns and Risk of Stroke Mortality

The other six articles evaluated four cohort studies (Ohsaki Cohort 1994, NIPPON DATA80, JACC, and JPHC) (Table 1). Of these six reports, three evaluated the Japanese dietary pattern scores by factor analysis [29,30,31], and the other three evaluated the Japanese dietary index scores using JFGS [34], JDI-8 [37], and low-salt Japanese diet score [32], respectively. Three articles on JPHC had similar follow-up periods [31,34,37], and each evaluated the scores by factor analysis [31], JFGS [34], and JDI-8 [37]. The pooled RR for stroke mortality compared with the highest to lowest adherence to the Japanese-style diet was 0.80 (95% CI: 0.69–0.93, *p* = 0.003, I^2^ = 66%, Egger’s test: *p* = 0.602), as shown in Table 2 and Figure 2B. The Japanese-style diet was significantly associated with reduced RR for stroke mortality.

#### 3.3.3. Japanese-Style Dietary Patterns and Risk of HD Mortality

The six articles evaluated four cohort studies (Ohsaki Cohort 1994, NIPPON DATA80, JACC, and JPHC) (Table 1). Of these six reports, three evaluated the Japanese dietary pattern scores by factor analysis [29,30,31], and the other three evaluated the Japanese dietary index scores using JFGS [34], JDI-8 [37], and low-salt Japanese diet score [32], respectively. Three JPHC studies had similar follow-up periods [31,34,37], and each evaluated the scores by factor analysis [31], JFGS [34], and JDI-8 [37]. The six articles evaluated the association with mortality risk for HD [31,34,37] or IHD [29,30,32]. The pooled RR for HD and IHD combined (HD/IHD) mortality in a comparison of the highest to lowest adherence to the Japanese-style diet was 0.81 (95% CI: 0.75–0.88, *p* < 0.001, I^2^ = 0%, Egger’s test: *p* = 0.544), as shown in Table 2 and Figure 2C. The Japanese-style diet was significantly associated with reduced RR for HD/IHD mortality.

#### 3.3.4. Characteristic Foods of the Japanese-Style Diet and Risk of CVD Mortality

##### Vegetables

**Figure 3:**
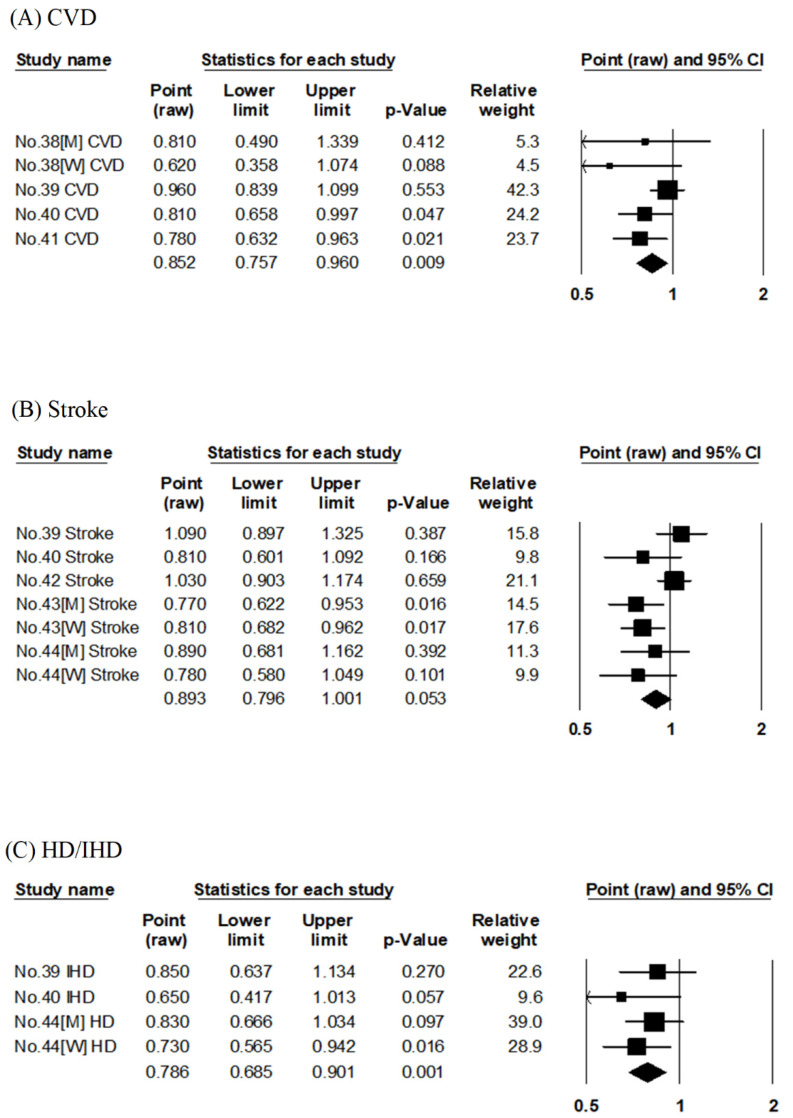
Meta-analysis of vegetable consumption and mortality risk. (**A**–**C**) show the association between vegetable consumption and CVD, stroke, and HD/IHD mortality risk, respectively. The area of each square is proportional to the study weight. Horizontal lines represent 95% confidence intervals. Diamonds represent pooled estimates from inverse-variance-weighted random-effects model. The number given in the study name indicates that of the cited reference [38,39,40,41,42,43,44]. CVD; cardiovascular disease, HD; heart disease, IHD; ischemic heart disease.

Four articles that investigated the data of three cohort studies (Takayama study, JACC, and NIPPON DATA80) evaluated the association between vegetable consumption and risk of CVD mortality [38,39,40,41] (Table S3). Two articles [40,41] on NIPPON DATA80 differed in the follow-up period, quantiles, and unit of food intake. The pooled RR for CVD mortality compared with the highest to lowest consumption of vegetables was 0.85 (95% CI: 0.76–0.96, *p* = 0.009, I^2^ = 21%, Egger’s test: *p* = 0.138), as shown in Table 2 and Figure 3A. A higher consumption of vegetables was significantly associated with reduced RR for CVD mortality.

Five articles evaluated the association between vegetable consumption and risk of stroke mortality [39,40,42,43,44]. These articles evaluated the results of five cohort studies (JACC, NIPPON DATA80, the six-prefecture cohort study, Hiroshima/Nagasaki Life Span Study, and JPHC). Regarding the pooled effect, the RR was 0.89 (95% CI: 0.80–1.001, *p* = 0.053, I^2^ = 52%, Egger’s test: *p* = 0.185), not indicating a significantly lower risk in the highest compared with the lowest vegetable consumption group (Table 2, Figure 3B).

Three articles evaluated the association between vegetable consumption and risk of HD/IHD mortality [39,40,44]. They evaluated the results of three cohort studies (JACC, NIPPON DATA80, and JPHC). The pooled RR for HD/IHD mortality compared with the highest to lowest consumption of vegetables was 0.79 (95% CI: 0.69–0.90, *p* < 0.001, I^2^ = 0%, Egger’s test: *p* = 0.349), as shown in Table 2 and Figure 3C. A higher consumption of vegetables was significantly associated with reduced RR for HD/IHD mortality.

##### Fruits

**Figure 4:**
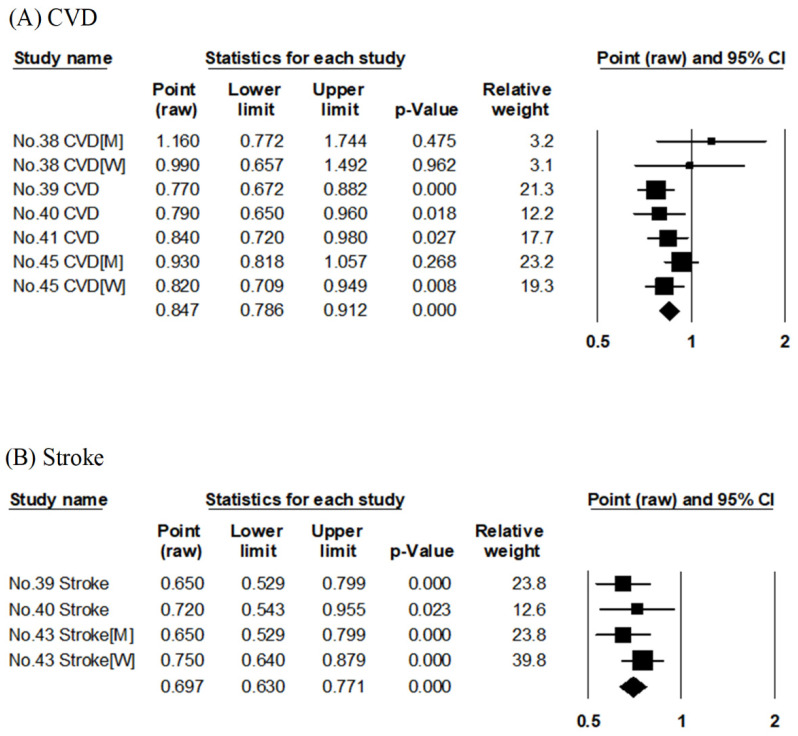
Meta-analysis of fruit consumption and mortality risk. (**A**,**B**) show the association between fruit consumption and CVD and stroke mortality risk, respectively. The area of each square is proportional to the study weight. Horizontal lines represent 95% confidence intervals. Diamonds represent pooled estimates from inverse-variance-weighted random-effects model. The number given in the study name indicates that of the cited reference [38,39,40,41,43,45]. CVD; cardiovascular disease.

Five articles evaluated the association between fruit consumption and risk of CVD mortality [38,39,40,41,45] (Table S3). They evaluated the results of three cohort studies (Takayama Study, JACC, and NIPPON DATA80). There were two reports each on NIPPON DATA80 [40,41] and JACC [39,45], but the reports of the same cohort differed in terms of the follow-up period, quantiles, and unit of food intake. The pooled RR for CVD mortality compared with the highest to lowest consumption of fruits was 0.85 (95% CI: 0.79–0.91, *p* < 0.001, I^2^ = 19%, Egger’s test: *p* = 0.296), as shown in Table 2 and Figure 4A. A higher consumption of fruits was significantly associated with reduced RR for CVD mortality.

Three articles evaluated the association between fruit consumption and risk of stroke mortality [39,40,43] (Table S3) using the results of three cohort studies (JACC, NIPPON DATA80, and Hiroshima/Nagasaki Life Span Study). The pooled RR for stroke mortality compared with the highest to lowest consumption of fruits was 0.70 (95% CI: 0.63–0.77, *p* < 0.001, I^2^ = 0%, Egger’s test: *p* = 0.605), as shown in Table 2 and Figure 4B. A higher consumption of fruits was significantly associated with reduced RR for stroke mortality.

In addition, two articles evaluated the association between fruit consumption and IHD mortality risk [39,40] (Table S3), in which no association was reported between fruit consumption and IHD mortality.

##### Fish

**Figure 5:**
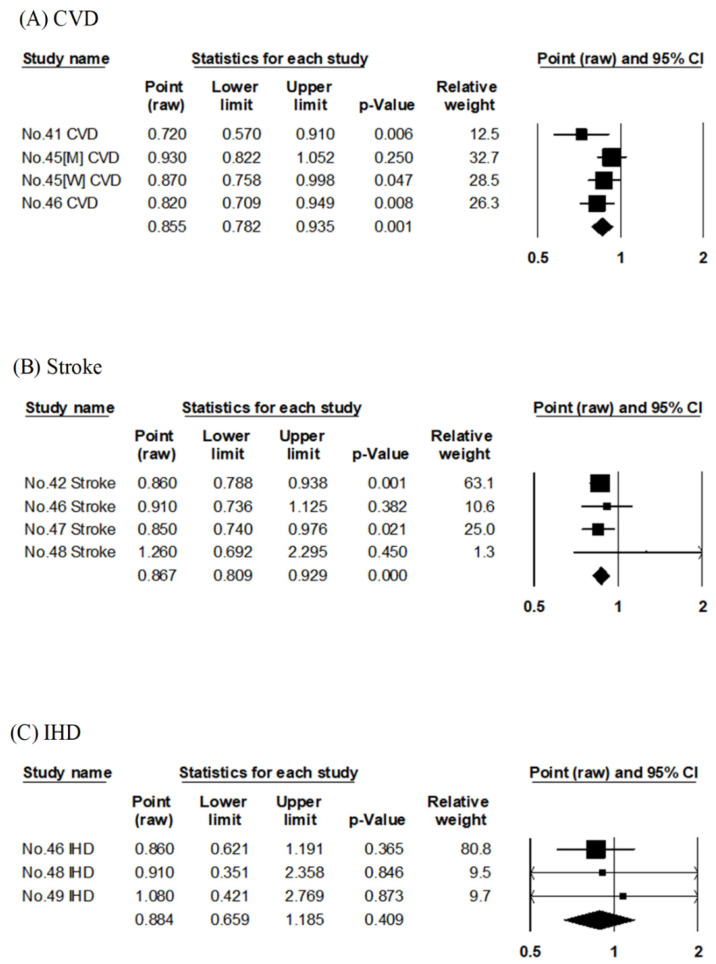
Meta-analysis of fish consumption and mortality risk. (**A**–**C**) show the association between fish consumption and CVD, stroke, and IHD mortality risk, respectively. The area of each square is proportional to the study weight. Horizontal lines represent 95% confidence intervals. Diamonds represent pooled estimates from inverse-variance-weighted random-effects model. The number given in the study name indicates that of the cited reference [41,42,45,46,47,48,49]. CVD; cardiovascular disease, IHD; ischemic heart disease.

Three publications evaluated the association between fish consumption and risk of CVD mortality [41,45,46] (Table S3) using the results of two cohort studies (NIPPON DATA80 and JACC). Two reports [45,46] on JACC differed in the follow-up period, quantiles, and unit of food intake. The pooled RR for CVD mortality compared with the highest to lowest consumption of fish was 0.86 (95% CI: 0.78–0.93, *p* < 0.001, I^2^ = 28%, Egger’s test: *p* = 0.082), as shown in Table 2 and Figure 5A. A higher consumption of fish was significantly associated with reduced RR for CVD mortality.

Four articles evaluated the association between fish consumption and risk of stroke mortality [42,46,47,48] (Table S3). They evaluated the results of four cohort studies (the six-prefecture cohort study, JACC, Hiroshima/Nagasaki Life Span Study, and NIPPON DATA80). The pooled RR for stroke mortality compared with the highest to lowest consumption of fish was 0.87 (95% CI: 0.81–0.93, *p* < 0.001, I^2^ = 0%, Egger’s test: *p* = 0.099), as shown in Table 2 and Figure 5B. A higher consumption of fish was significantly associated with reduced RR for stroke mortality.

Three articles evaluated the association between fish consumption and risk of IHD mortality [46,48,49] (Table S3) using the results of three cohort studies (JACC, NIPPON DATA80, and JPHC). The pooled RR for IHD mortality compared with the highest to lowest consumption of fish was 0.88 (95% CI: 0.66–1.19, *p* = 0.409, I^2^ = 0%, Egger’s test: *p* = 0.380), and there was no association (Table 2, Figure 5C).

Regarding other diseases related to CVD, the death risk from pulmonary embolism was reduced in the group with the highest fresh fish consumption (0.17, 95% CI: 0.05–0.64) [50], and the death risk from aortic disease was increased in people who rarely ate fish (1.93, 95% CI: 1.13–3.31, *p* for trend = 0.009) [51] (Table S3).

#### Soy Products

Four articles examined the association between soy product consumption and risk of CVD mortality [39,52,53,54] (Table S3) using the results of four cohort studies (JACC, Takayama Study, Jichi Medical School Cohort Study, and JPHC). The pooled RR for CVD mortality compared with the highest to lowest consumption of soy products was 0.94 (95% CI: 0.87–1.02, *p* = 0.137, I^2^ = 0%, Egger’s test: *p* = 0.640), and there was no association between the consumption of soy products and risk of CVD mortality (Table 2, Figure S1).

There were also no associations between the consumption of soy products and risk of stroke and HD mortality [39,54]. However, the mortality risk was reduced for ischemic CVD (cerebral and myocardial infarction combined) in women (0.31, 95% CI: 0.13–0.74, *p* for trend = 0.006) [55] (Table S3). Moreover, the study suggested that there was no association between bean intake and risk of CVD mortality [39].

Regarding soy products, tofu [54,56], miso soup [42,55], miso [54], and natto [54,57] were evaluated for their associations with the risk of CVD mortality (Table S3). In women, higher tofu consumption was found to reduce the risk of death from cerebral hemorrhage [56] (0.35, 95% CI: 0.14–0.85, *p* for trend = 0.030). Another article on tofu [54] reported a reduced risk of HD mortality in men (0.74, 95% CI: 0.61–0.90, *p* for trend = 0.005). Miso soup consumption was not found to be associated with the mortality risk of stroke [42] and ischemic CVD [55]. There was also no relationship between miso intake and risk of death from CVD, stroke, or HD [54]. Higher natto consumption reduced the mortality risk of HD in men (0.71, 95% CI: 0.57–0.88, *p* for trend = 0.01) and stroke in women (0.67, 95% CI: 0.50–0.89, *p* for trend = 0.02) [54]. Higher natto consumption also resulted in reduced mortality risk of stroke and its subtype, cerebral infarction (stroke: 0.68, 95% CI: 0.52–0.88, *p* for trend = 0.004, cerebral infarction: 0.67, 95% CI: 0.47–0.95, *p* for trend = 0.03) [57].

##### Green Tea

**Figure 6:**
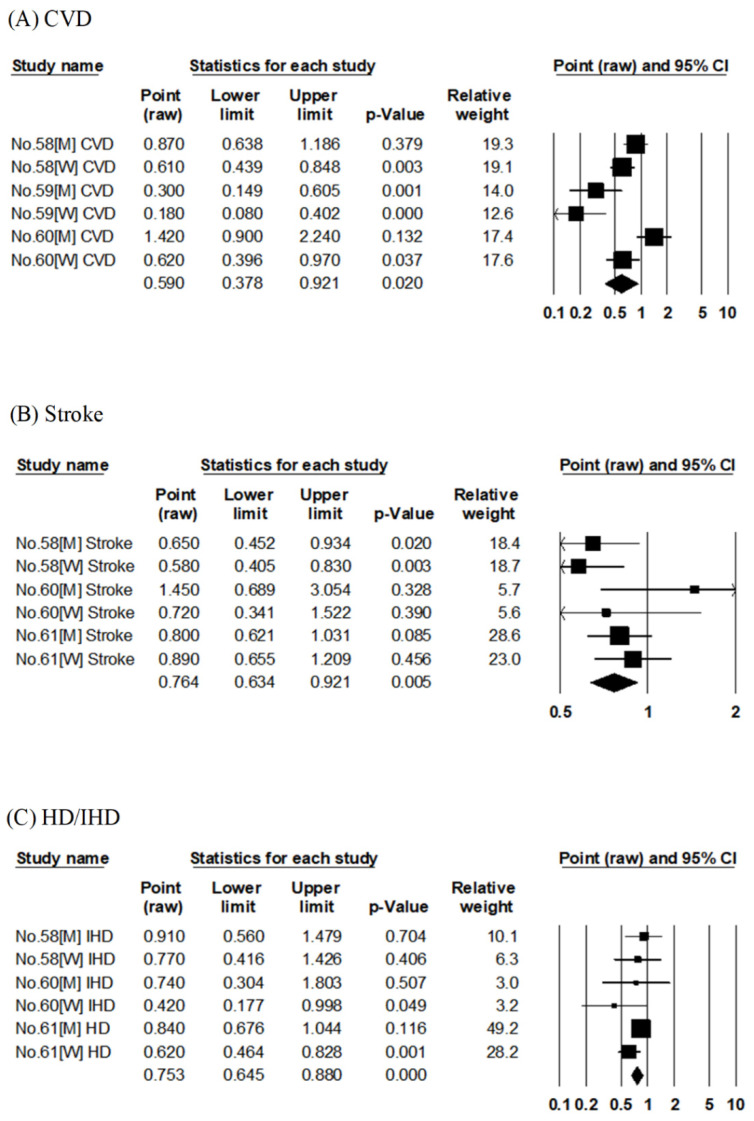
Meta-analysis of green tea consumption and mortality risk. (**A**–**C**) show the association between green tea consumption and CVD, stroke, and HD/IHD mortality risk, respectively. The area of each square is proportional to the study weight. Horizontal lines represent 95% confidence intervals. Diamonds represent pooled estimates from inverse-variance-weighted random-effects model. The number given in the study name indicates that of the cited reference [58,59,60,61]. CVD; cardiovascular disease, HD; heart disease, IHD; ischemic heart disease.

Three articles evaluated the association between green tea consumption and risk of CVD mortality [58,59,60] (Table S3) using the results of three cohort studies (Ohsaki Cohort 1994, Shizuoka Elderly Cohort, and JACC). The pooled RR for CVD mortality in a comparison of the highest to lowest consumption of green tea was 0.59 (95% CI: 0.38–0.92, *p* = 0.020, I^2^ = 83%, Egger’s test: *p* = 0.182), indicating a reduced risk of CVD mortality in the group with the highest consumption of green tea (Table 2, Figure 6A).

Three articles examined the association between green tea consumption and risk of stroke mortality [58,60,61] (Table S3) using the results of three cohort studies (Ohsaki Cohort 1994, JACC, and JPHC). The pooled RR for stroke mortality in a comparison of the highest to lowest consumption of green tea was 0.76 (95% CI: 0.63–0.92, *p* = 0.005, I^2^ = 28%, Egger’s test: *p* = 0.691), indicating a reduced risk of stroke mortality in the group with the highest green tea consumption (Table 2, Figure 6B).

Three publications examined the association between green tea consumption and risk of HD or IHD mortality [58,60,61] (Table S3) using the results of three cohort studies (Ohsaki Cohort 1994, JACC, and JPHC). One study evaluated the association with the risk of HD mortality [61], and two studies evaluated the association with the risk of IHD mortality [58,60]. The pooled RR for HD/IHD mortality in a comparison of the highest to lowest consumption of green tea was 0.75 (95% CI: 0.65–0.88, *p* < 0.001, I^2^ = 1%, Egger’s test: *p* = 0.515), indicating a reduced risk of HD/IHD mortality in the group with the highest consumption of green tea (Table 2, Figure 6C).

##### Milk or Dairy Products

Three articles evaluated the association between the consumption of milk or milk and dairy products and the risk of CVD mortality [45,62,63] (Table S3). They evaluated the results of two cohort studies (JACC and NIPPON DATA80). Two reports on JACC [45,63] differed in terms of follow-up period and quantiles. The pooled RR for CVD mortality in a comparison of the highest to lowest consumption of milk or dairy products was 0.93 (95% CI: 0.87–0.998, *p* = 0.045, I^2^ = 37%, Egger’s test: *p* = 0.963), indicating a reduced risk of CVD mortality in the group with the highest consumption of dairy products (Table 2, Figure S2A).

Three articles examined the association between the intake of milk or dairy products and the risk of stroke mortality [42,47,62] (Table S3) using the results of three cohort studies (the six-prefecture cohort study, Hiroshima/Nagasaki Life Span Study, and NIPPON DATA80). The pooled RR for stroke mortality in a comparison of the highest to lowest consumption of dairy products was 0.81 (95% CI: 0.75–0.88, *p* < 0.001, I^2^ = 17%, Egger’s test: *p* = 0.583), indicating a reduced risk of stroke mortality in the group with the highest consumption of dairy products (Table 2, Figure S2B). Furthermore, one report [62] evaluated the association between the consumption of dairy products and the risk of IHD mortality, but no association was reported.

##### Rice

Three articles examined the association between rice consumption and risk of stroke mortality [64,65,66] (Table S3) using the results of three cohort studies (Takayama Study, JACC, and JPHC). The pooled RR for stroke mortality in a comparison of the highest to lowest consumption of rice was 1.02 (95% CI: 0.89–1.17, *p* = 0.807, I^2^ = 0%, Egger’s test: *p* = 0.808), and there was no association between rice consumption and risk of stroke mortality (Table 2, Figure S3). There was also no association between rice consumption and risk of IHD mortality [66] (1.08, 95% CI: 0.84–1.38, *p* for trend = 0.56). However, it was suggested that higher rice consumption reduced the IHD mortality risk only in men [65] (0.70, 95% CI: 0.49–0.99, *p* for trend = 0.02).

##### Meat

Three articles examined the association between meat consumption and risk of stroke mortality [42,47,67] (Table S3) using the results of three cohort studies (the six-prefecture cohort study, Hiroshima/Nagasaki Life Span Study, and JACC). The pooled RR for stroke mortality in a comparison of the highest to lowest consumption of meat was 0.91 (95% CI: 0.76–1.09, *p* = 0.319, I^2^ = 63%, Egger’s test: *p* = 0.069), and there was no association between meat consumption and risk of stroke mortality (Table 2, Figure S4). One of these articles reported risk reduction of IHD mortality only in men [67] (0.66, 95% CI: 0.45–0.97, *p* for trend = 0.015).

##### Other Foods

Six articles evaluated the association between the consumption of other foods and CVD mortality [42,47,68,69,70,71] (Table S3). One article that examined the association between seaweed consumption and risk of CVD mortality reported that higher consumption significantly reduced the risk only in women [68] (0.72, 95% CI: 0.55–0.95, *p* for trend = 0.001). Among three [47,69,70] articles that evaluated the relationship between egg consumption and risk of CVD mortality, only one article reported that higher egg consumption was associated with a decreased risk of stroke mortality [47] (0.70, 95% CI: 0.51–0.95, *p* for trend = 0.185).

One article evaluated the association between pickle consumption and risk of stroke mortality [42]. Higher pickle consumption was associated with a reduced risk of stroke mortality (0.91, 95% CI: 0.83–0.99), but it did not affect the mortality risk of intracerebral hemorrhage or cerebral infarction. Moreover, one article reported that increasing dietary diversity reduced the risk of CVD mortality only in women [71] (0.66, 95% CI: 0.51–0.86, *p* for trend = 0.009).

#### 3.3.5. Nutrients of the Japanese-Style Diet and Risk of Cardiovascular Disease Mortality

##### Salt (Sodium)

**Figure 7:**
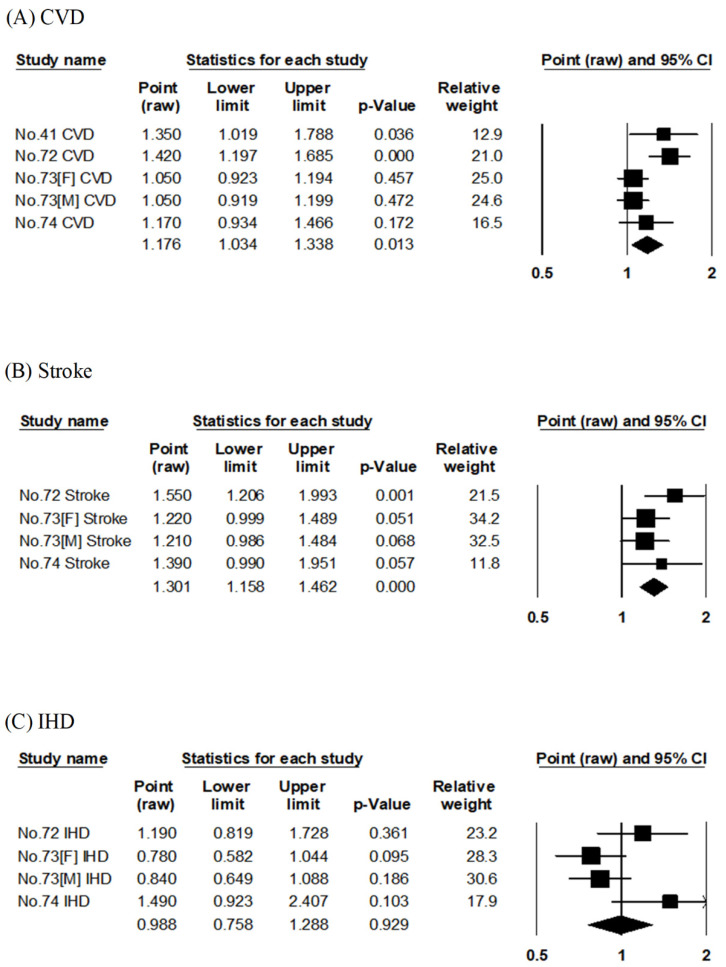
Meta-analysis of salt consumption and mortality risk. (**A**–**C**) show the association between salt consumption and CVD, stroke, and IHD mortality risk, respectively. The area of each square is proportional to the study weight. Horizontal lines represent 95% confidence intervals. Diamonds represent pooled estimates from inverse-variance-weighted random-effects model. The number given in the study name indicates that of the cited reference [41,72,73,74]. CVD; cardiovascular disease, IHD; ischemic heart disease.

Four articles examined the association between salt intake and risk of CVD mortality [41,72,73,74] (Table S3) using the results of two cohort studies (NIPPON DATA80 and JACC). Two reports on NIPPON DATA80 [41,74] and the other two reports on JACC [72,73] differed in quantiles, unit of salt consumption, and period of follow-up. The pooled RR for CVD mortality compared with the highest to lowest consumption of salt was 1.18 (95% CI: 1.03–1.34, *p* = 0.013, I^2^ = 63%, Egger’s test: *p* = 0.243), indicating significant risk increment in the group with higher salt intake (Table 2, Figure 7A).

Three articles evaluated the association between salt intake and risk of stroke and IHD mortality [72,73,74] (Table S3). They evaluated the results of two cohort studies (JACC and NIPPON DATA80). Two studies on JACC [72,73] differed in the quantiles of salt consumption and duration of follow-up. The pooled RR for stroke mortality in a comparison of the highest to lowest consumption of salt was 1.30 (95% CI: 1.16–1.46, *p* < 0.001, I^2^ = 0%, Egger’s test: *p* = 0.358), indicating significant risk increment in the group with higher salt intake (Table 2, Figure 7B). The pooled RR for IHD mortality compared with the highest to lowest salt consumption was 0.99 (95% CI: 0.76–1.29, *p* = 0.929, I^2^ = 59%, Egger’s test: *p* = 0.065), not affecting the risk of mortality (Table 2, Figure 7C).

In addition, a 2g increase in salt intake per 1000 kcal of energy intake was reported to increase the mortality risks of CVD, stroke, and IHD [74] (CVD: 1.11, 95% CI: 1.03–1.19; *p* for trend = 0.007; stroke: 1.12, 95% CI: 1.00–1.25; *p* for trend = 0.044; and IHD: 1.25, 95% CI: 1.08–1.44; *p* for trend = 0.002) (Table S3).

These results suggested that higher salt intake counteracted the risk reduction effects of the characteristic foods such as vegetables, fruits, fish, and green tea.

##### Protein

**Figure 8:**
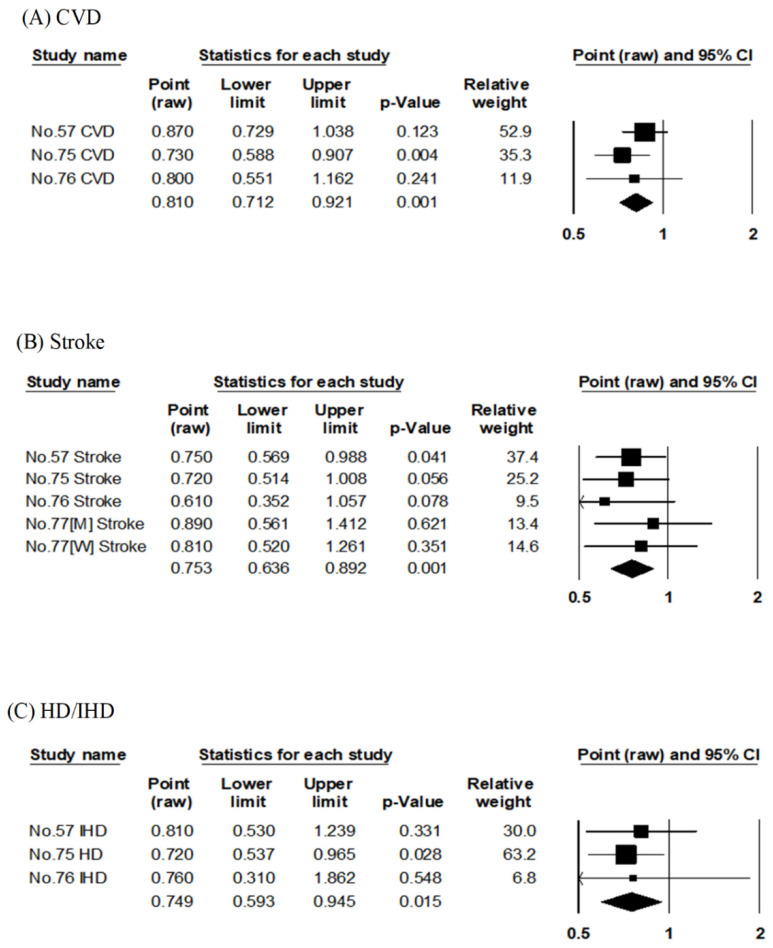
Meta-analysis of plant-derived protein consumption and mortality risk. (**A**–**C**) show the association between plant-derived protein consumption and CVD, stroke, and HD/IHD mortality risk, respectively. The area of each square is proportional to the study weight. Horizontal lines represent 95% confidence intervals. Diamonds represent pooled estimates from inverse-variance-weighted random-effects model. The number given in the study name indicates that of the cited reference [55,75,76,77]. CVD; cardiovascular disease, HD; heart disease, IHD; ischemic heart disease.

Three articles examined the association between plant-derived protein intake and risk of CVD and HD/IHD mortality [57,75,76] (Table S3) using the results of three cohort studies (Takayama Study, JPHC, and NIPPON DATA90). The pooled RRs for CVD and HD/IHD mortality compared with the highest to lowest plant-derived protein intake were respectively 0.81 (95% CI: 0.71–0.92, *p* = 0.001, I^2^ = 0%, Egger’s test: *p* = 0.772) and 0.75 (95% CI: 0.59–0.95, *p* = 0.015, I^2^ = 0%, Egger’s test: *p* = 0.696), indicating risk reduction of both mortality in the highest intake group (Table 2, Figure 8A,C).

Four articles evaluated the association between plant-derived protein intake and risk of stroke mortality [57,75,76,77] (Table S3) using the results of three cohort studies (Takayama Study, JPHC, and NIPPON DATA90). Two reports on the Takayama study used soy [57] and vegetable-derived proteins [77] as plant-derived protein, respectively. The pooled RR for stroke mortality in a comparison of the highest to lowest intake of plant-derived proteins was 0.75 (95% CI: 0.64–0.89, *p* = 0.001, I^2^ = 0%, Egger’s test: *p* = 0.967), indicating a reduced risk of stroke mortality in the group with the highest consumption of plant-derived proteins (Table 2, Figure 8B).

The study on the intake of plant-derived protein also reported that a 1% increment of plant protein intake (% energy) reduced the mortality risk of CVD (0.86, 95% CI: 0.75–0.99) and cerebral hemorrhage (0.58, 95% CI: 0.35–0.95) [76] (Table S3). Animal protein intake did not affect the risk of CVD, stroke, and HD mortality [75,77] (Table S3). However, a higher animal protein intake reduced the risk of cerebral infarction mortality (0.47, 95% CI: 0.24–0.92, *p* for trend = 0.0212). In contrast, plant-derived protein intake had no association with cerebral infarction mortality risk [78] (Table S3).

##### Carbohydrate

Four articles evaluated the association between CVD mortality and intake of carbohydrates other than dietary fiber [64,79,80,81] (Table S3). Of the three articles on the Takayama Study, two evaluated the association with the risk of CVD mortality [79,80], and one evaluated the risk of stroke mortality [64]. In men, higher starch consumption reduced the risk of CVD mortality (0.62, 95% CI: 0.45–0.86, *p* for trend = 0.008), whereas higher consumption of saccharides (monosaccharides and disaccharides) increased that risk [79] (1.39, 95% CI: 1.08–1.78, *p* for trend = 0.001). In women, consuming a diet with a higher glycemic index increased the mortality risk of CVD (1.56, 95% CI: 1.15–2.13, *p* for trend = 0.007) [80] and stroke (2.09, 95% CI: 1.01–4.31, *p* for trend = 0.10) [64]. However, another cohort study on NIPPON DATA 80 reported no association between starch consumption and risk of mortality from CVD [81]. Two cohort studies also reported no association between carbohydrate intake and mortality risk of CVD and stroke [64,81].

##### Dietary Fiber

**Figure 9:**
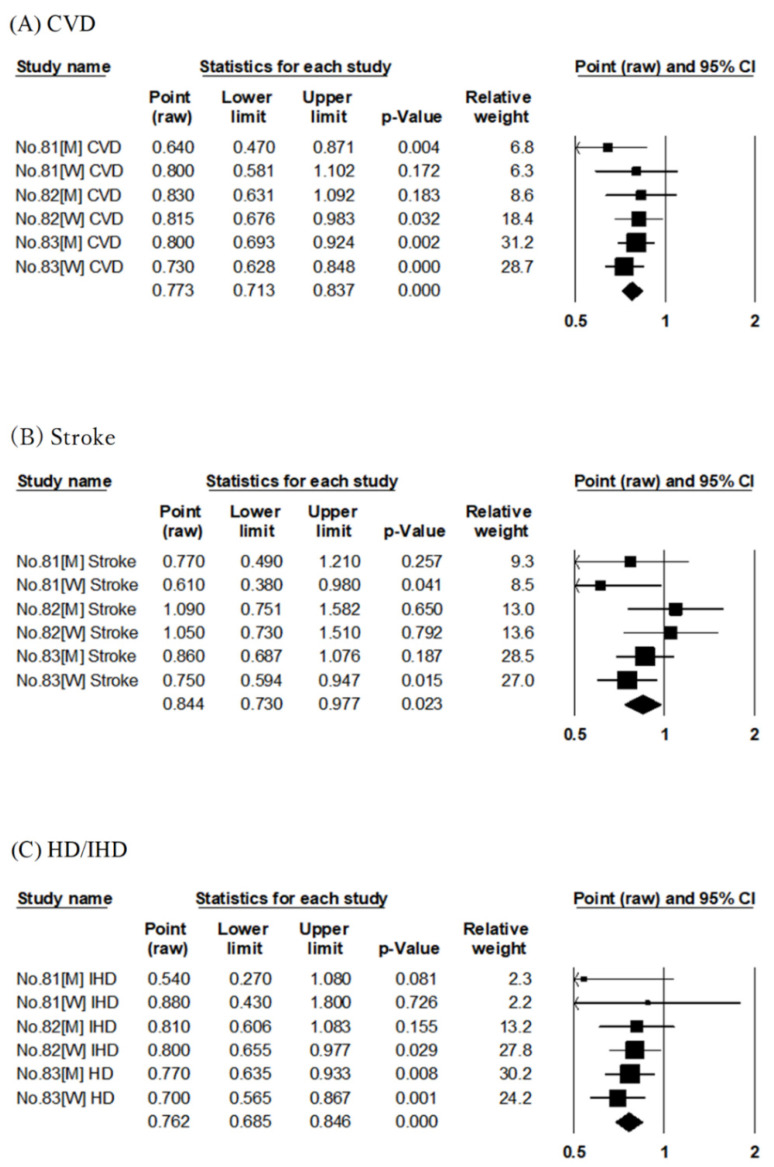
Meta-analysis of dietary fiber consumption and mortality risk. (**A**–**C**) show the association between dietary fiber consumption and CVD, stroke, and HD/IHD mortality risk, respectively. The area of each square is proportional to the study weight. Horizontal lines represent 95% confidence intervals. Diamonds represent pooled estimates from inverse-variance-weighted random-effects model. The number given in the study name indicates that of the cited reference [81,82,83]. CVD; cardiovascular disease, HD; heart disease, IHD; ischemic heart disease.

Three articles examined the association between dietary fiber intake and risk of CVD, stroke, and HD or IHD mortality [81,82,83] using the results of three cohort studies (NIPPON DATA80, JACC, and JPHC) (Table S3). The pooled RRs for CVD, stroke, and HD/IHD mortality comparing the highest and lowest dietary fiber intake groups were 0.77 (95% CI: 0.71–0.84, *p* < 0.001, I^2^ = 0%, Egger’s test: *p* = 0.794), 0.84 (95% CI: 0.73–0.98, *p* = 0.023, I^2^ = 19%, Egger’s test: *p* = 0.941), and 0.76 (95% CI: 0.69–0.85, *p* < 0.001, I^2^ = 0%, Egger’s test: *p* = 0.665), indicating a reduced mortality risk of all diseases in the highest intake group (Table 2, Figure 9A–C).

##### Fat

Three articles evaluated the association between saturated fatty acid intake and risk of CVD mortality [84,85,86] (Table S3) using the results of two cohort studies (JACC and Takayama Study). Two studies on JACC differed in terms of the unit of saturated fatty acid intake and the follow-up period [84,86]. The pooled RR for CVD mortality comparing the highest and lowest saturated fatty acid intake was 0.95 (95% CI: 0.84–1.07, *p* = 0.365, I^2^ = 34%, Egger’s test: *p* = 0.350), and there was no association (Table 2, Figure S5).

A higher intake of saturated fatty acid was reported to reduce the risk of stroke mortality (0.69, 95% CI: 0.53–0.89, *p* for trend = 0.004) but was not associated with the risk of IHD mortality (0.93, 95% CI: 0.65–1.35, *p* for trend = 0.86) [84]. Higher consumption of animal fats reportedly reduced the risk of cerebral infarction mortality [78] (0.36, 95% CI: 0.17–0.76, *p* for trend = 0.0032). However, there was no association between total fat intake and the risk of CVD mortality [85,86].

## 4. Discussion

According to the recent World Health Statistics, Japan has the highest life expectancy in the world. Japanese-style dietary patterns and characteristic Japanese food intake were suggested to contribute to disease prevention and lifespan extension by large cohort studies in Japan; however, the epidemiologic evidence is much less than that of the Mediterranean diet. In studies outside Japan, a review utilizing several meta-analysis studies demonstrated that a Mediterranean diet reduces the risk of CVD [87]. “Prudent pattern” and “Healthy pattern” reportedly reduced the risk of CVD, stroke, and HD, whereas the “Western pattern” and “Unhealthy pattern” are characterized by meat and processed meat and do not reduce that risk [88]. Therefore, we conducted a systematic review on the relationship between the Japanese-style diet or characteristic Japanese food intake and the mortality risk for major diseases such as CVD, stroke, and HD.

Based on a systematic review on the Japanese-style diet, Suzuki et al. [12] reported that the highly ranked components of the Japanese-style diet were soybean products, seafood, vegetables, rice, miso soup, seaweed, pickles, green tea, and fruits. The studies included in our review used the Japanese dietary index score calculated by index methods and the Japanese dietary pattern score calculated by factor analysis as adherence to the Japanese-style diet. Therefore, both scores reflect the characteristic elements of the Japanese-style diet. Among the characteristic elements of Japanese food, vegetables, fruits, beans, and fish, are similar to “Prudent pattern,” “Healthy pattern,” and “Mediterranean dietary pattern” in Western studies. On the other hand, soybean products, which are major sources of plant-derived protein, calcium, and the phytoestrogen isoflavones for Japanese people, green tea, and rice are characteristic ingredients of the Japanese-style diets.

The meta-analysis in this review revealed that the Japanese-style diet reduced the mortality risk of CVD, its constituents, stroke, and HD. It has been reported that the higher the adherence to the Japanese-style diet, the lower the risk factors such as blood pressure and serum cholesterol [89,90,91], and the better intake of nutritional components and trace elements [92,93]. These factors may contribute to the CVD risk reduction by the Japanese-style diet.

In this review, increased intake of vegetables and fruits was found to reduce the risk of CVD mortality. These results support the results of a meta-analysis performed by Wang X. et al. [94], Aune D. et al. [95], and Zurbau A. et al. [96]. Vegetables and fruits are considered common risk-reducing elements for CVD worldwide without restriction to the Japanese-style diet. Tsubota-Utsugi et al. [97] showed that increased fruit consumption reduces the risk of developing hypertension. Okayama et al. [98] reported that a smaller dietary sodium-to-potassium ratio is associated with a lower risk of CVD mortality. Dietary fiber is also known to lower serum cholesterol levels [99,100]. Vegetables and fruits are the leading intake sources of dietary fiber and potassium intake, and these foods may contribute to the risk reduction of CVD mortality. Increased intake of vegetables and fruits may reduce the CVD mortality risk exacerbated by increased salt intake.

In this review, increased fish consumption reduced the mortality risk of CVD and stroke. This result supports the result of a meta-analysis study conducted by Jayedi et al. [101]. In contrast, no reduction in IHD mortality risk was detected in the meta-analysis of the present review. However, we do not exclude the possibility of reducing the mortality risk of IHD by fish consumption. In an article [49] used for pooled analysis, increased intakes of fish and n-3 polyunsaturated fatty acids, the primary components of fish oils, were found to reduce the risk of developing nonfatal IHD. Furthermore, a meta-analysis of studies conducted both in and outside Japan reported that higher fish consumption reduced the mortality risk of IHD [102]. DHA/EPA present in fish oil lowers serum triglycerides levels [103,104]. This lowering action may contribute to the reduced risk of CVD. We expect that further observational studies in Japan will provide an apparent conclusion.

In Japan, the daily intake of soybeans and soy products is 59 g [22], and soybeans are the leading source of protein. No association was found between soy product intake and mortality risk of CVD in the meta-analysis of this review. However, the consumption of soy products such as tofu and natto correlated negatively with CVD and HD mortality risk [54,56,57]. Nozue et al. [105] reported that consumption of fermented soybean products reduced the risk of developing hypertension. Uemura et al. [106] also indicated that the intake of fermented soy products and soy isoflavones negatively correlated with arterial stiffness in Japanese men. These effects may contribute to the reduced risk of CVD in soy products. Furthermore, a case-control study conducted outside Japan reported a negative association between the consumption of soy products and mortality risk of CVD and IHD [107]. This review showed that increased intake of plant-derived protein reduced the mortality risk of CVD, stroke, and HD. Accumulating results suggest the possible contribution of soy products to the reduction of CVD mortality risk. On the other hand, in Japan, the intake of vegetables and fruits that contribute to risk reduction has not reached the target intake, and that of fish and soybeans is on a downward trend. These intake trends may weaken the risk-reducing effect of soy products on CVD.

Regarding green tea, a higher consumption reduced the mortality risk of stroke and HD in a pooled analysis of eight cohort studies conducted in Japan [108]. This review also revealed a similar reduction in stroke and HD mortality risk. Green tea and epigallocatechin gallate present in green tea reportedly lower LDL-cholesterol [109,110], LDL-oxidation [111,112], and blood pressure [113,114] levels. These lowering effects may play a role in reducing the mortality risk of CVD.

A meta-analysis of prospective cohort studies conducted in and outside Japan reported negative associations between dairy consumption and risk of CVD and stroke. However, no association occurred with the risk of IHD [115,116]. Another report [117] also indicated that higher consumption of dairy products and milk reduced the mortality risk of stroke, whereas higher consumption of milk increased the mortality risk of IHD. In this review, increased consumption of milk or dairy products reduced the mortality risk of CVD and stroke. Umesawa et al. [118,119] showed that increased intake of dairy-derived calcium reduced the risk of stroke. Dairy-derived calcium may contribute to the risk reduction by milk and dairy products.

In this review, a higher salt intake increased the mortality risk of CVD and stroke. Aburto et al. [120] reported that reduced salt (sodium) consumption lowered blood pressure. He et al. [121] also reported that in the UK, a reduction in salt consumption lowered blood pressure and the mortality for stroke and IHD. These results indicate that a balanced diet of vegetables, fruits, and fish and decreasing salt intake may be higher risk reduction.

In this review, we found no association between rice intake and mortality risk of CVD. A higher sugar intake and consumption of diets with a high glycemic index increased the mortality risk of CVD [79,80]. However, this review revealed that higher dietary fiber intake reduced the mortality risk of CVD, stroke, and HD/IHD. From the viewpoint of carbohydrate intake, sufficient dietary fiber intake becomes essential, and it may be necessary to consume cereals such as brown rice and barley and vegetables, fruits, beans, potatoes, and seaweeds.

Meat intake in Japan is known to be much lower than that of the U.S.A. or E.U., although the intake and the energy intake from fat have increased noticeably since 1965. This review showed no association between the intake of saturated fatty acids and CVD mortality risk. However, increased consumption of total fat and saturated fatty acids reduced the risk of intracerebral hemorrhage [122]. Furthermore, increased saturated fatty acid intake reduced the mortality risk of intracerebral hemorrhage and cerebral infarction but did not affect the risk of HD [84]. Moderate increases in meat and fat intake may have contributed to the reduced risk of stroke. In contrast, there is a positive association between serum cholesterol level and saturated fatty acids intake [123] and IHD mortality risk [124]. It is necessary to be aware of changes in IHD risk, as significant increases in meat and fat intake could increase the risk of IHD. In a meta-analysis of prospective cohort studies conducted in Asian countries, including Japan, higher red meat consumption reduced the risk of CVD mortality in men [125]. In the past, meat consumption was low in Asian countries, so the increased intake might improve the nutritional balance and reduce the risk.

These reports indicated that a balanced intake of total fat, saturated fatty acids, and polyunsaturated fatty acids is essential for CVD risk reduction. Moreover, a balanced protein intake derived from animals and plants is essential for CVD risk reduction. Considering the primary intake sources of fat and protein, a balanced intake of meat (quantity and type), fish, and soy products decreases the mortality risk of stroke and HD and consequently reduces the overall risk of CVD.

Meta-analyses conducted between CVD and stroke mortality risk and the primary food elements suggested that the risk reduction by the Japanese-style diet is attributable to counteracting the risk increment by higher salt intake throughout risk reduction due to vegetables, fruits, and fish. These results suggest that the following food intake directions are essential for reducing the CVD mortality risk: (1) increasing the intake of vegetables and fruits not reaching the target amount, (2) increasing the intake of fish, indicating a downward trend, (3) decreasing the intake of meat instead, (4) decreasing the intake of salt, and (5) complementary ingestion of soy products, dairy products, and green tea. These directions of risk reduction support the report of Tada et al. [126] that maintaining adequate calories, increasing fish and vegetable consumption, decreasing refined carbohydrate and animal fat consumption, and decreasing salt consumption may be beneficial strategies for preventing IHD and stroke.

Sugawara et al. [127] conducted a 4-week interventional study comparing the effect of diet in 1975 with that in 2015 for men and women in their twenties. They reported that the group consuming the 1975-type diet, compared with the group consuming the 2015-type diet, showed lower body fat, lower serum triglyceride and LDL-cholesterol levels, and higher serum HDL-cholesterol levels. These results suggest that recent diet has increased the CVD risk. A characteristic feature of the 1975-type diet is nutritionally lower fat-derived energy intake. Moreover, compared with the 2015-type diet, it is characterized by (1) a greater number of foodstuffs, (2) more cooking methods such as boiling, steaming, and eating raw, (3) increased consumption frequency of soybean products, seafood, vegetables (pickles), fruits, green tea, seaweeds, and mushrooms, (4) increased use of soup stocks “dashi” and fermented seasonings, and (5) a set of rice and soup [127]. This report suggests the importance of efficient intake of foods known to reduce risk but not reach the target intake and reduced intake of risk-increasing foods. Therefore, the form and ingenuity for consuming foodstuffs leading to risk reduction may be the basis for a healthy Japanese-style diet.

In this review, we showed the most recent evidence on the mortality risk reduction of Japanese-style diet or characteristic Japanese food intake on CVD, stroke, or HD. Our results also suggest a way to improve dietary life for Japanese people. In the future, we expect a new large-scale cohort study matching the current dietary habits of Japan. By comparing and examining the results of previous studies, we expect to find a healthier dietary pattern and reaffirm the goodness of the Japanese-style diet “Washoku”.

## 5. Limitations

There are two primary limitations in this research. First, as numerous cohort studies have conducted diet surveys between 1980 and 1990s, their results may vary from the current pattern of the Japanese diet. Murakami et al. [128] investigated changes in the diet pattern based on the National Health and Nutrition Examination Survey from 2003 to 2015. They reported a decrease in the “plant foods and fish pattern” scores and an increase in “bread food and dairy products pattern” and “animal foods and oil-fat pattern” scores. They also found that the decrease in “plant foods and fish pattern” scores was a common phenomenon across all generations stratified and that the increase in “bread food and dairy pattern” and “animal foods and oil-fat pattern” scores occurred among older generations. Second, the meta-analysis in this review used articles that evaluated the same cohort study by different methods. Because of the small number of studies on most items, it was impossible to perform a pooled analysis by extracting only one article per cohort. Therefore, we performed a meta-analysis using all the studies adopted.

## 6. Conclusions

This review clarified the association between Japanese-style diet/characteristic Japanese foods and CVD mortality. Higher adherence to the Japanese-style diet reduced the mortality risk of CVD, stroke, and HD/IHD. Furthermore, a higher intake of vegetables, fruits, fish, green tea, milk and dairy products, dietary fiber, and plant-derived protein reduced the mortality risk of one or more of CVD, stroke, and HD/IHD. Our results suggest that the Japanese-style diet/characteristic Japanese foods reduce CVD mortality in the Japanese population.

## Figures and Tables

**Table 1:** Prospective cohort studies on Japanese-style dietary pattern and the CVD mortality risk (Hazard ratios, risk ratios, and 95% confidence intervals).

First Author, Publication Year (Reference No.)	Population	Sample Size (sex)	Age Range (years)	Follow-Up Period (years)	Diet Assessment Method	Dietary Pattern Assessment Method	Intervention: Control	Outcome (ICD Code) No. of Deaths	Hazard Ratio/Risk Ratio (95%CI)	Factors Adjusted for in Analyses
Shimazu, 2007 [29]	Ohsaki Cohort 1994 Study	40,547 (both)	40–79	7	FFQ	Factor analysis Japanese dietary pattern: high intake of soy products, fish, seaweeds, vegetables, fruits, green tea	Higest:Lowest scores quartile	CVD (I00–99) Both: 801 Stroke (I60–69) Both: 432 Cerebral infarction (I63) Intracerebral hemorrhage (I61) IHD (I20–25) Both: 181	0.74 (0.59–0.91) 0.64 (0.48–0.86) 0.60 (0.37–0.99) 0.60 (0.36–1.03) 0.82 (0.52–1.29)	Age, sex, smoking status, walking duration, education, total energy intake, body mass index, history of hypertension
Maruyama, 2013 [30]	JACC	26,598 (men) 37,439 (women)	40–79	Median: 12.6	FFQ	Factor analysis Vegetable pattern: high intake of fresh fish, vegetables, fungi, potatoes, algae, tofu, fruits	Higest:Lowest scores quintile	CVD (I01–99) Men: 1240, Women: 1071 Stroke (I60–69) Men: 578, Women: 499 IHD (I20–25) Men: 272, Women: 207	Men 0.93 (0.78–1.13) 1.13 (0.85–1.51) 0.73 (0.49–1.08) Women 0.82 (0.67–1.00) 0.91 (0.68–1.22) 0.67 (0.43–1.06)	Age, BMI, smoking category, walking time, hours of sports, education, perceived mental stress, sleep duration, total energy intake and history of hypertension and diabetes
Nanri, 2017 [31]	JPHC	81,720 (both)	45–74	Mean: 14.8	FFQ	Factor analysis Prudent dietary pattern: high intake of vegetables, fruit, soy products, potatoes, seaweed, mushrooms, fish	Higest:Lowest scores quartile	CVD (I00–99) Both: 2813 Stroke (I60–69) Both: 1096 HD (I20–52) Both: 1478	0.72 (0.64–0.79) 0.63 (0.53–0.75) 0.75 (0.66–0.87)	Age, sex, study area, body mass index, smoking status, total physical activity, history of diabetes mellitus, history of hypertension, and total energy intake
Nakamura, 2009 [32]	NIPPON DATA80	9086 (both)	30 years or older	19	FFQ	Index score (Reduced-Salt JDS)	Higest:Lowest scores tertile	CVD (I00–99) Both: 654 Stroke (I60–69) Both: 299 IHD (AMI; I21–22) Both: 131	0.80 (0.66–0.96) 0.75 (0.56–0.99) 0.84 (0.55–1.27)	Age, sex, BMI, smoking, hypertension, diabetes
Oba, 2009 [33]	Takayama Study	13,355 (men) 15,724 (women)	35 years or older	7.3 (9/1992–12/1999)	FFQ	Index score (JFGS)	Higest:Lowest scores quartile	CVD (I00–99) Men: 308, Women: 327	Men 1.06 (0.78–1.45) Women 0.76 (0.56–1.04)	Age, body mass index, smoking status, physical activity, education, history of hypertension and diabetes, women’s menopausal status
Kurotani, 2016 [34]	JPHC	79,594 (both)	45–75	Mean: 14.9	FFQ	Index score (JFGS)	Higest:Lowest scores quartile	CVD (I00–99) Both: 2560 Stroke (I60–69) Both: 1005 HD (I20–52) Both: 1.342	0.84 (0.73–0.96) 0.78 (0.63–0.97) 0.84 (0.70–1.02)	Age, sex, and public health centre area, BMI, smoking status, total physical activity, history of hypertension, history of diabetes, history of dyslipidaemia, coffee consumption, green tea consumption, occupation
Okada, 2018 [35]	JACC	23,162 (men) 34,232 (women)	40–79	Median: 18.9(men) 19.4(women)	FFQ	Index score (JFS)	Higest:Lowest scores quintile	CVD (I05–99) Men: 1674, Women: 1734	Men 0.89 (0.76–1.04) Women 0.66 (0.56–0.77)	Age, geographical region, BMI, education duration, smoking status, alcohol drinking status, sports habits, sleeping duration, history of hypertension and diabetes, total energy intake
Abe, 2020 [36]	Ohsaki Cohort 1994 Study	14,764 (both)	40–79	20	FFQ	Index score (JDI)	Higest:Lowest scores quartile	CVD (I00–99) Both: 1352	0.96 (0.82–1.13)	Sex, education level, smoking, alcohol drinking, time spent walking, history of disease, energy intake, BMI
Matsuyama, 2021 [37]	JPHC	92,969 (both)	45–74	Median: 18.9	FFQ	Index score (JDI-8)	Higest:Lowest score quartile	CVD (I00–99) Both: 4990 Stroke (I60–69) Both: 1950 HD (I20–52) Both: 2600	0.89 (0.80–0.99) 0.89 (0.75–1.05) 0.89 (0.77–1.03)	Age, sex, study area, BMI, smoking status, alcohol drinking, total physical activity, medication, occupation, total energy intake

CVD; cardiovascular disease, HD; heart disease, IHD; ischemic heart disease, AMI; acute myocardial infarction.

## Supplementary Materials

The following supporting information can be downloaded at: https://www.mdpi.com/2072-6643/14/10/2008/s1, Table S1: PRISMA checklist; Table S2: Search strategy; Table S3: Prospective cohort studies on the intake of foods or nutrition and the CVD mortality risk (Hazard ratios, risk ratios, and 95% confidence intervals); Table S4: Quality assessment sheet for each article; Table S5. Body of evidence for the results of each meta-analysis; Figure S1: Meta-analysis of soy products consumption and CVD mortality risk; Figure S2: Meta-analysis of milk and dairy products consumption and mortality risk; Figure S3: Meta-analysis of rice consumption and stroke mortality risk; Figure S4: Meta-analysis of meat consumption and stroke mortality risk; Figure S5: Meta-analysis of saturated fatty acids consumption and CVD mortality risk.
